# *DUX4*-rearranged B-ALL: deciphering a biological and clinical conundrum

**DOI:** 10.1038/s41375-025-02758-5

**Published:** 2025-09-12

**Authors:** Jack Bakewell, Anthony V. Moorman, Sarra L. Ryan

**Affiliations:** Translational and Clinical Research Institute, Newcastle University Centre for Cancer, Faculty of Medical Sciences, Newcastle upon Tyne, UK

**Keywords:** Haematological cancer, Cancer genetics

## Abstract

The *DUX4* gene, located within repetitive subtelomeric arrays on chromosomes 4 and 10, plays a critical role in early embryogenesis and has been implicated in several human diseases, including facioscapulohumeral muscular dystrophy (FSHD) and cancer. In B-cell acute lymphoblastic leukemia (B-ALL), *DUX4* rearrangements (*DUX4*-r) define a distinct genomic subtype affecting 5–10% of cases, which is more frequent among older children and teenagers. These rearrangements produce truncated DUX4 proteins with neomorphic transcriptional activity, resulting in aberrant gene expression programs and alternative splicing that disrupt normal B-cell precursor development. Patients with *DUX4*-r B-ALL often present with poor initial treatment responses, though they typically achieve excellent long-term survival rates with intensive chemotherapy regimens. The cryptic nature of *DUX4* rearrangements has historically posed significant challenges to accurate detection, but recent advancements in next-generation sequencing technologies, including RNA and long-read sequencing, and improved immunophenotyping strategies—such as the use of CD371 as a surrogate marker—are enhancing diagnostic accuracy. This review explores the genetic and biological features of *DUX4* and its rearrangements, shedding light on their role in leukemogenesis and associated clinical outcomes. Additionally, we highlight emerging technologies that enable the detection of *DUX4*-r and discuss their implications for clinical use and research. An improved understanding of *DUX4* biology and its oncogenic potential may pave the way for novel treatment strategies, ultimately improving outcomes for patients with *DUX4*-r B-ALL.

## Introduction

The *DUX4* gene has been implicated in human disease development, including leukemogenesis [1]. In 2016, genetic abnormalities of *DUX4* were first discovered within a specific subtype of childhood acute lymphoblastic leukaemia (ALL), termed *DUX4*-r ALL [2]. *DUX4*-r ALL comprises 5–10% of B-cell precursor cases and tends to occur most commonly in older children and teenagers, a group of patients usually associated with poorer treatment response when compared to younger children [3]. In the majority of instances, the driving mutation is the translocation of *DUX4* to the *IGH* locus in immature B-cells, leading to the expression of a truncated DUX4 protein [2]. The location of *DUX4* within highly repetitive sequence on the subtelomeres of chomosomes 4 and 10 has made such rearrangements extremely challenging to genetically detect and biologically characterise, complicated further by variant isoform transcription and recombination between alleles of homologous sequence [4]. As a result, the mechanism by which *DUX4*-r induces leukemogenesis is unclear. In this review, we discuss what is known about the genetic composition of the *DUX4* loci and the emerging genomic and biological features of wild-type (wt) and aberrant *DUX4* expression in normal development and disease, focusing on leukemogenesis. *DUX4*-r exhibits a unique gene expression profile among ALL subtypes, driven by neomorphic transcriptional activity distinct from that of wt DUX4 protein [5]. Recent studies have made significant strides in characterising IGH::DUX4 cofactors and downstream pathway activation, creating promising new avenues of research for therapeutic invervention.

A metadata analysis of clinical features shows that *DUX4*-r patients have a specific demographic profile and excellent survival rates (>95% in children, >80% in adults), despite over half of patients responding poorly to initial induction therapy [6, 7]. There is uncertainty over whether the excellent survival rates are driven by the more intensive treatment received due to the association of measurable residual disease (MRD) at day 28 [8]. The accurate detection of these patients at diagnosis is currently a conundrum for most genetic testing laboratories. This review also summarises the technologies and laboratory approaches that have been assessed and used for identifying *DUX4*-r patients, and we discuss their broader utility for prospective screening. Recent studies have begun to unravel the role of *DUX4*-r in leukaemia development and treatment response, but future efforts must rely on accurate detection in prospective clinical trials and international collaboration to determine the origin, behaviour and outcomes of *DUX4*-r in leukaemia.

## DUX4 genetics and wildtype function

### Genetics

The double homebox (*DUX*) genes encode a family of transcription factors (TFs) that play key roles in the early embryonic development of eutherian mammals [9]. In humans, these consist of the three paralogues *DUXA, DUXB* and *DUX4* [10], of which only *DUX4* has been extensively characterised due to its roles in facioscapulohumeral muscular dystrophy (FSHD) [11] and multiple cancers [1, 12, 13].

*DUX4* is expressed at two independent loci, 4q35 (also known as D4Z4) and 10q26, where it is nested within each 3.3 kb repeat unit of a macrosatellite array [14]. These arrays exhibit >99% sequence homology and recombination events between them are frequent [15]. The only consistent difference is the deletion of the 6-mer sequence “CGCCTC” in 10q26 repeats compared to D4Z4 [16]. Both arrays typically consist of 11–100 repeat units and only the final *DUX4* copy is transcribed [11].

**Fig. 1:**
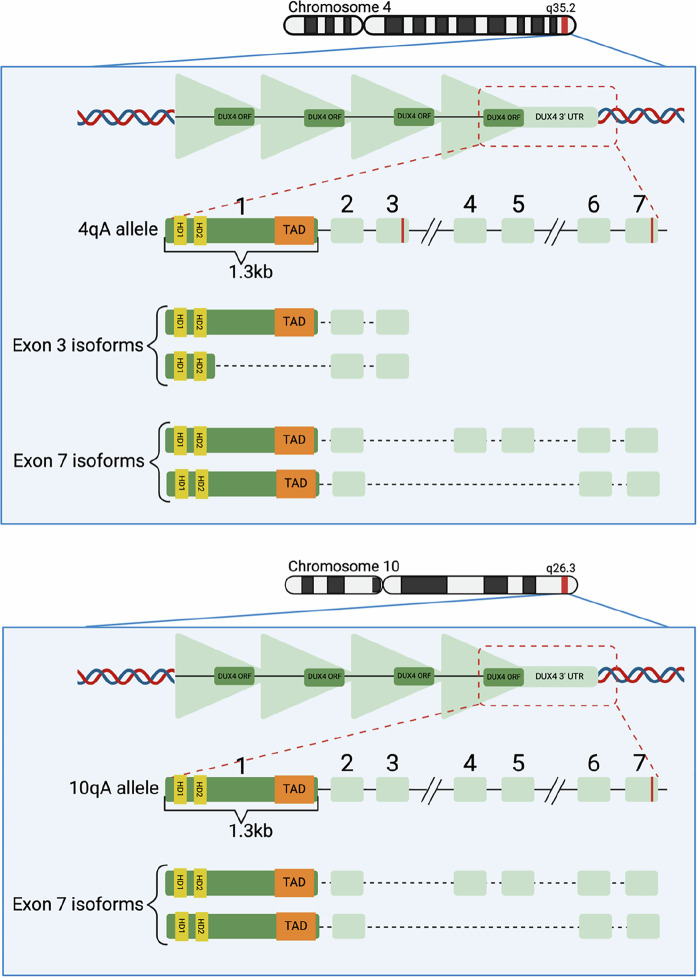
Macrosatellite arrays in the subtelomeric regions from chromosomes 4 (top) and 10 (bottom). Each contains the *DUX4* open reading frame (ORF), of which only the distal unit is transcribed. The seven non-coding exons identified in A alleles are shown, along with polyadenylation signals (red lines) and known transcript isoforms. Created in BioRender. Ryan, S. (2025) https://BioRender.com/3zn0iuh.

Each macrosatellite repeat unit includes a 1.4 kb intronless open reading frame (ORF) comprising the entire DUX4 protein coding sequence, while at the distal end of each ORF is a single non-coding exon. At both *DUX4* loci, there are two major alleles that are equally frequent in the general population: A and B [17]. Downstream of the final ORF, A alleles exhibit five non-coding exons that contribute to tissue-specific splice isoforms (Fig. 1) [17, 18]. These exons are missing on both B alleles and it is not currently known how B-allele transcripts are stabilised [18].

All full-length *DUX4* transcripts produce a protein at whose N-terminus are two homeoboxes that serve as its DNA binding domain [19], while at its C-terminus is a transactivation domain (TAD) which recruits histone acetyltransferases (HATs) for gene activation [20]. The amino acids spanning from the second homeobox to the TAD are intrinsically disordered, such that targeted deletion of this sequence does not affect *DUX4* expression levels or function [21]. A single short-length transcript has been identified, arising from the A allele of the D4Z4 locus, which gives rise to a DUX4 protein without a TAD [18].

### *DUX4* in healthy tissue

*DUX4* expression primarily occurs in the pre-implantation embryo, where it is tightly regulated by telomeric remodelling during embryo cleavage [22], acting as a pioneer factor in the first main wave of zygotic genome activation [23]. Hundreds of genes are directly activated by DUX4 [24], which collectively ensure genome stability, clearance of maternal transcripts and proteins, and the establishment of totipotentiality [25, 26]. Full-length chromosome 4 and 10 transcripts have also been detected in equal amounts in the germline cells of the testes [18] and in T cells of the thymus [27], though their function in these tissues is unclear.

## DUX4 in non-leukaemic disease

### *DUX4* in FSHD

FSHD was the first disorder to be associated with aberrant expression of *DUX4*. It is the third most common type of muscular dystrophy, with a worldwide prevalence of 1 in 8000 to 20,000 individuals, depending on ancestry [28]. Symptoms tend to onset before the age of twenty and are characterised by a progressive deterioration of skeletal muscles in the face, upper body and lower extremities [29]. No effective treatment options currently exist; however, life expectancy is not shortened compared to the general population [30].

FSHD has only been observed in carriers of the D4Z4 4qA allele, present in around half of the European population [17]. This allele contains a non-canonical polyadenylation signal (PAS) at exon 3, which serves to efficiently stabilise transcripts, leading to a cytotoxic build-up of DUX4 protein and its targets [11, 17].

**Fig. 2:**
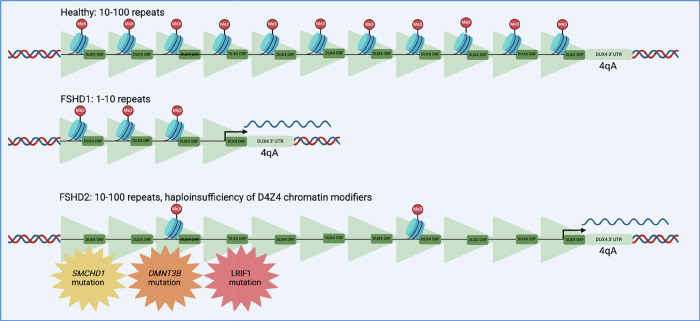
The genomic subtypes of facioscapulohumeral muscular dystrophy (FSHD). While FSHD1 involves contraction of the D4Z4 array to 1–10 repeat units, mutations of D4Z4 chromatin modifier genes are implicated in FSHD2. Both require the 4qA allele and lead to aberrant expression of *DUX4* in skeletal muscle due to hypomethlyation of the *DUX4* open reading frame (ORF) from the final repeat unit. Created in BioRender. Ryan, S. (2025) https://BioRender.com/ylvekuu.

Cases can be categorised into two genetic subtypes: FSHD1 and FSHD2 (Fig. 2). The pathogenic variant in FSHD1 is the contraction of D4Z4 to 1-10 repeat units and is evident in 95% of cases [31]. D4Z4 contraction causes remodelling of chromatin, resulting in significant loss of repressive H3K9me3 methylation in the distal repeat unit and *DUX4* gain-of-function, specifically in skeletal myocytes [31].

The disease mechanism of FSDH2 is haploinsufficiency of genes associated with D4Z4 methylation [32]. Unlike FSHD1 wherein only the distal repeat unit is hypomethylated, FSHD2 is associated with global hypomethylation that impacts the whole D4Z4 array, though it does not result in increased levels of *DUX4* expression or a more severe phenotype compared with FSHD1 [33]. However, these mutations can act as disease modifiers of D4Z4 contraction, resulting in worse symptoms compared to cases that exhibit only the latter [34].

Despite the ability of DUX4 to facilitate cancer progression, cancer risk in FSHD is not elevated above that of the general population [35], though recent findings suggest that gastrointestinal cancers in particular may be more prevalent among FSHD adults (>40 years) than in non-affected adults [36].

### *DUX4* in non-leukaemic cancers

*DUX4* expression in advanced-stage solid cancers correlates with reduced expression of chemokines and MHC class 1 genes, and inhibition of T cell recruitment to tumour sites [13, 37, 38]. These findings indicate that *DUX4* facilitates a tumour microenvironment that is less conducive to effective immune responses, promoting cancer progression and potential resistance to immunotherapy. It is not known why *DUX4* is preferentially expressed in advanced-stage cancers, though transient expression of *DUX4* transcriptional regulators *DPPA2* and *DPPA4* has been detected in cases, along with loss-of-function mutations in likely *DUX4* repressors [13, 37], and it is possible that increased telomeric shortening derepresses *DUX4* in a manner similar to that observed in cleavage-stage embryos [39].

*DUX4* gene fusions have been identified as driver mutations in sarcomas, a rare group of malignancies that develop in connective tissue and bone [40]. There are more than 150 known subtypes of sarcoma, the majority (87%) of which occur in soft tissue of the extremities, trunk, head and neck [40]. They account for <1% of malignant cancers in adults but are significantly more common in children and adolescents, comprising 15-20% of paediatric malignancies [40].

**Fig. 3:**
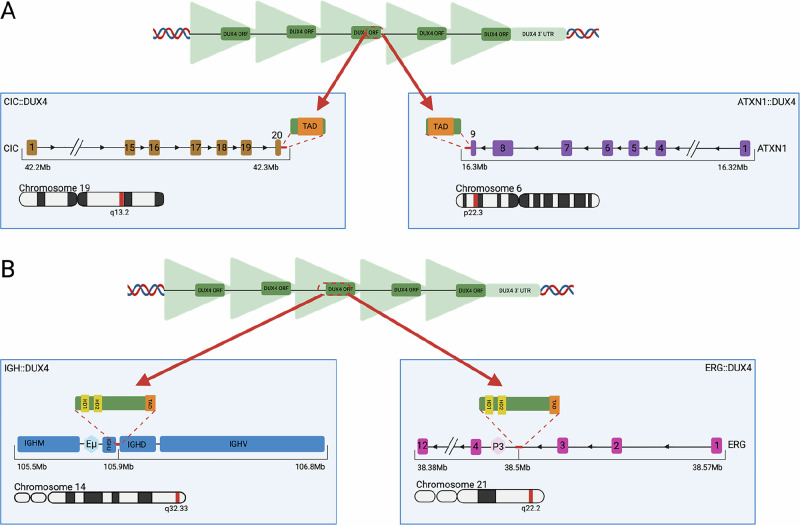
Common breakpoints of DUX4 rearrangements in cancer. Breakpoints of *CIC*-rearranged sarcoma (**A**) and *DUX4* rearrangements identified in *DUX4*-r B-ALL (**B**). Translocation of *DUX4* sequence is represented by red arrows in both diagrams. In B-ALL, breakpoints at *DUX4* loci occur in the TAD, giving rise to proteins in which the TAD is truncated. Enhancer (Eμ at *IGH*) or promotor (likely P3 at *ERG*) regions are ‘hijacked’ to induce aberrant *DUX4* expression in B cell precursors. In *CIC*-rearranged sarcoma, only the *DUX4* TAD is translocated, fusing with the final exons of *CIC* or *ATXN1*, resulting in a chimeric protein that activates typically repressed *CIC* target genes in soft tissue of the central nervous system. Base pair coordinates were extracted from human genome build hg38. Created in BioRender. Ryan, S. (2025) https://BioRender.com/1pqisgg.

*DUX4* rearrangements have been implicated in ~1% of round cell sarcomas, a high-grade subset characterised by a round, small and undifferentiated cellular morphology [12], where its fusion partner is the Capicua gene *CIC* [41]*. CIC*-rearranged sarcomas typically affect children and young adults (median age 25–35 years), and are notable for their aggressive course and poor outcomes [42]. Capicua is a transcriptional repressor that plays a role in neuronal differentiation, immune cell development and embryogenesis [43]. It also acts a tumour suppressor, in part by silencing the polyomavirus enhancer activator 3 (*PEA3*) family of oncogenic transcription factors [44]. *CIC::DUX4* produces a chimeric protein in which the C-terminal end of Capicua is fused with the DUX4 C-terminal TAD [41] (Fig. 3A), the addition of which results in the acetylation of Capicua targets by p300/CBP, dramatically increasing their expression [41].

Two cases of round cell sarcomas have been reported in the CNS that share the gene expression profile of the *CIC*-rearranged subtype, and yet were negative for *CIC* alterations [45, 46]. In both, the *DUX4* TAD was fused in-frame with the final exon of *ATXN1*, producing a protein that, similar to *CIC::DUX4*, contains almost the entire Ataxin 1 sequence flanked by the *DUX4* TAD (Fig. 3A). Ataxin 1 interacts directly with Capicua to stabilise its repressor complex [47], suggesting that the addition of the TAD may activate Capicua targets in the same manner as *CIC::DUX4*.

Finally, a *DUX4* fusion event has also been implicated in embryonal rhabdomyosarcoma (ERMS), another soft tissue sarcoma in which the affected cells morphologically resemble the developing skeletal myocytes of the embryo [48]. Fusion events are very rare in ERMS, for which driver events typically involve chromosomal gains [48]. In the single reported case, the patient showed a normal karyotype (46, XX) but carried a translocation of *DUX4* to the *EWSR1* gene locus at 22q12 [49]. Interestingly, some undifferentiated round cell sarcomas and RMS blasts have been found to originate from a common mesenchymal stem cell [50, 51]. The occurrence of *DUX4* rearrangements among these sarcoma subtypes may indicate that *DUX4* could have an oncogenic potential that is specific to this stem cell type.

## *DUX4*-rearranged B-ALL

### Description of *DUX4* rearrangements

**Fig. 4:**
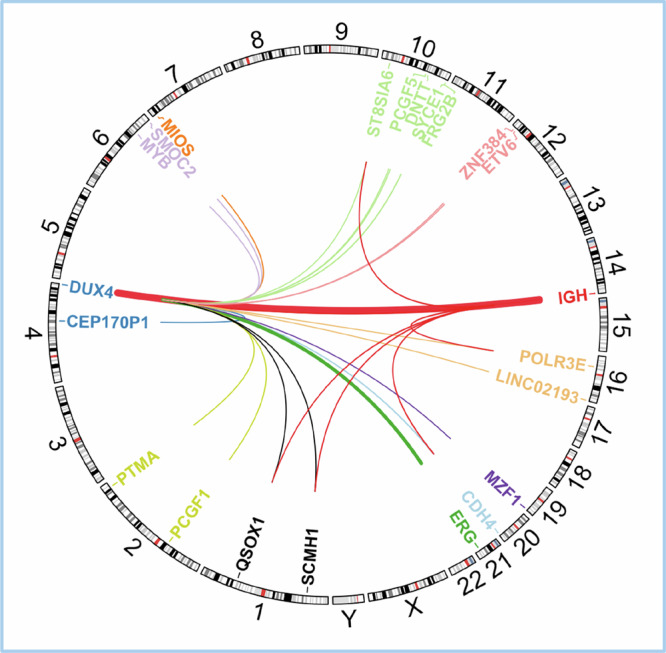
*DUX4*-r gene partners reported in the literature [1–3, 7, 52, 57, 59, 61, 63, 64, 73, 74, 86, 87, 98, 100–104]. Lines between genes represent translocation, with the colour of lines corresponding to destination chromosomes. In the case of triple fusions (e,g. *IGH::QSOX1::DUX4*), both *DUX4* and the second gene partner fuse at the *IGH* locus, represented by two lines, first from *DUX4* to the second gene parter, then to *IGH*. Among 695 *DUX4*-r cases for which a gene partner has been identified in the literature, 663 (95%) are *IGH::DUX4* (excluding triple fusions), 7 (1%) are *ERG::DUX4* and 3 (0.4%) are *ETV6::DUX4*. All others are single cases. Gene coordinates are derived from the human genome build hg38.

In *DUX4*-r B-ALL, repeat units from the D4Z4 or 10q26 loci are typically inserted out-of-frame into the immunoglobulin heavy locus (*IGH*) or – more rarely - the ETS-related gene (*ERG*) [3, 52], though a wide range of other gene partners have been identified in around 4.6% of *DUX4-*r cases (Fig. 4). The rearrangement involves insertion of a partial repeat copy, or one complete and one partial copy, of *DUX4* in either strand orientation [52]. Reciprocal events, where *IGH* sequences are inserted into the *DUX4* locus, can occur in some cases [53]. ‘Triple fusions’ have been detected in five cases, whereby *DUX4* is fused with sequence from a third gene partner at the *IGH* locus, and it is conceivable that some non-recurrent *DUX4* gene partners occur in triple fusion events wherein the *IGH* component was not detected [1].

*IGH* breakpoints for *IGH::DUX4* rearrangements mostly occur in a 3kb sequence overlapping the diversity (*IGH-D*) and joining (*IGH-J*) segments of *IGH*, near to the Eμ transcriptional enhancer (Fig. 3B) [2]. These segments undergo somatic rearrangement in B-cell precursors, wherein RAG endonuclease complexes induce double-stranded breaks at recombination signal sequences (RSS) [54]. DNA repair enzymes add random nucleotides and the processed ends are ligated [54]. RSS-like sequences have been detected at the breakpoints of translocation events in the *IGH* locus [55], suggesting the possibility that substrate-selection errors of RAG complexes are implicated in the insertion of *DUX4* sequence.

*DUX4* breakpoints are enriched in the 5’ region upstream of the *DUX4* ORF and in the 3’ end of the ORF itself [2, 56]. Truncation of the *DUX4* C-terminus is therefore observed in almost all *IGH::DUX4* transcripts, leading to the replacement of up to twenty amino acids with *IGH* sequence. In the NALM6 cell line, for instance, an endogenous insertion of a full and partial repeat unit from the 10q26 array into *IGH* leads to a transcript involving only the partial unit, in which the final sixteen amino acids of wt *DUX4* are replaced by those of the *IGH-D* sequence, including a PAS [53].

Phased RNA-seq data from NALM6 cells also showed allelic imbalance from the *IGH* locus, with the wt *IGH* allele being more active than the *IGH::DUX4* rearrangement, indicating that the translocation took place on the silenced *IGH* allele [53]. During B-cell development, *IGH* allelic exclusion ensures that each B-cell produces a single type of antibody by first silencing both alleles through hypermethylation, then selectively activating one during RAG-mediated somatic rearrangement. While it is unknown whether the silenced *IGH* allele is affected in all *DUX4*-r cases, it is possible that excess expression of *DUX4* hinders leukemogenesis due to cytotoxicity, though there is no evidence that overexpression of *IGH::DUX4* is pro-apoptotic [5].

In *ERG::DUX4*, D4Z4/10q26 repeat units are inserted into *ERG* intron 3 (Fig. 3B) [52, 57]. Interestingly, this intron has been found to be a hotspot for double-strand breaks induced by aberrant activity of the androgen receptor complex [58], suggesting that *DUX4* insertions at *ERG* take place through an alternate mechanism to that at the *IGH* locus. Similar to *IGH::DUX4*, however, transcripts typically result in a truncated C-terminal *DUX4* protein and have the same gene expression profile [52], indicating that downstream leukaemic activity is identical.

### Secondary genomic alterations

Intragenic *ERG* deletions (*ERG*del) are a critical secondary event in *DUX4*-r B-ALL and occur almost exclusively in this subtype [59], with multiple studies showing that >95% of B-ALL cases with *ERG*del harbour a *DUX4* rearrangement [2, 60, 61]. Indeed, *ERG*del was detected and proposed as a subgroup of BCP-ALL before *DUX4* rearrangement events were discovered [62]. Typically, *ERG* deletions are focal, most commonly involving exons 3–7 or 3–9, and are thought to be secondary events arising from the binding of the DUX4 rearranged protein, but not wt *DUX4*, to an alternative transcription initiation site within intron 6 of *ERG* [2, 60, 61].

DUX4 rearranged proteins can also induce transcription of alternative *ERG* transcripts, *ERG*alt, originating from a non-canonical exon. The most common isoform results from splicing that joins the novel exon to exon 7, producing a truncated protein that has reduced transactivation ability and acts as a competitive inhibitor of wildtype ERG [2].

*ERG*alt transcripts contain exons that are not present in the deleted *ERG* allele, and are detectable in cases that do not carry the *ERG*del [63], indicating that it arises from the unaffected allele. Moreover, *ERG*alt transcripts are more abundant in *ERG*del cases, suggesting that activity of the DUX4 rearranged protein is positively correlated with the likelihood of *ERG*del [63].

Interestingly, mouse models have revealed a role for isoform-specific *ERG* in oncogenesis. While animals expressing full-length *ERG* in bone marrow succumb to an aggressive erythromegakaryoblastic leukaemia, those expressing *ERG*alt develop lymphoid leukaemias with longer latency [2], suggesting that *ERG*alt may itself contribute to leukemogenesis in *DUX4*-r cases.

**Fig. 5:**
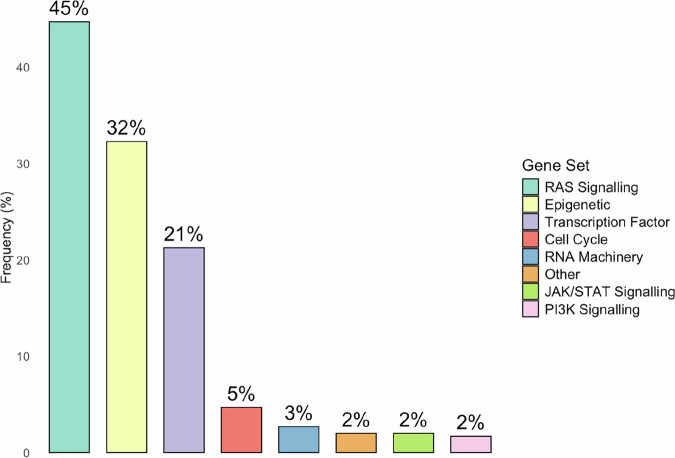
Frequency of non-silent gene mutations, grouped by gene sets. Findings were meta-analyzed using data from seventeen studies in which non-silent mutations were investigated [1, 3, 52, 57, 59, 63, 64, 81, 86, 87, 98–105]. Gene sets were created by combining those used in Zhang et al. [2] and Brady et al. [64], which assessed mutations across all B-ALL subtypes.

Mutational analyses of *DUX4*-r cases showed a mean of 17.5 non-silent somatic sequence mutations per case (range 2–42) [2], and a median of 0.25 SNVs per Mb [64]. A minority of *DUX4*-r patients show enrichment for the mutational signature associated with reactive oxygen species [64]. Across all currently published studies that have investigated the incidence of non-silent mutations in *DUX4*-r, 44.7% of cases carry mutations affecting genes involved in the RAS/MAPK pathway, while 32.3% carry mutations in epigenetic pathway genes, and 21.3% of cases carry mutations affecting transcription factor genes (Fig. 5).

**Fig. 6:**
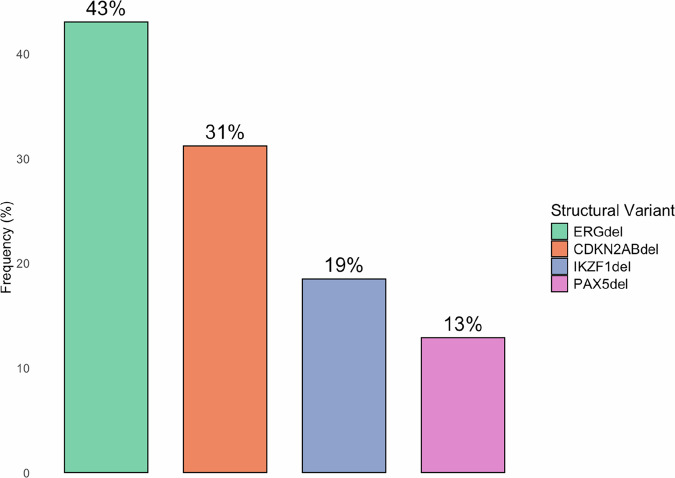
Frequency of intragenic structural variants (SV) in DUX4-r cases. Findings were metanalysed using data from twenty-one studies which screened for at least one of the four specified deletion events. [1, 2, 7, 52, 57, 59, 61, 63–65, 73, 74, 86, 87, 100–106].

*DUX4*-r B-ALL is characterized by a low incidence of other chromosomal alterations compared to other B-ALL subtypes [65], though *DUX4*-r carriers of *ERG*del have been shown to harbour significantly more intragenic structural alterations than non-carriers [63]. Across published studies that have analysed intragenic structural variants in *DUX4*-r, the most common events are *ERG* deletion (43.1% of cases), *CDKN2A/B* deletion (31.2%), *IKZF1* deletion (18.5%), and *PAX5* deletion (12.9%) (Fig. 6).

### Transactivation and gene expression profile

While IGH::DUX4 binds 97% of the same targets as wt DUX4 in NALM6 [56, 66] ten-fold fewer genes are transcriptionally activated by IGH::DUX4 compared to the wild-type [66]. Therefore, it has been suggested that the truncation of the C-terminal attenuates the ability of DUX4 rearranged protein to recruit p300/CEBP for transactivation, behaving as a less efficient isoform of wt DUX4 [53]. However IGH::DUX4 can recruit cofactors that are not involved in wt DUX4 transactivation and initiate novel mechanisms of alternative splicing, giving rise to a distinct gene expression profile that is unique among B-ALL subtypes [61].

Despite their identical DNA-binding domains, ChIP-seq studies against IGH::DUX4 have shown that active target sites are more enriched in intronic and intergenic sites compared to wt DUX4, while comparisons of transcriptional activity have found minimal overlap between both upregulated and downregulated gene sets [5, 66]. Target genes of wt DUX4 that play key roles in embryo development, such as *ZSCAN4, DUXA* and *RFPL4A*, are not activated by IGH::DUX4, which instead upregulates genes enriched in cell adhesion, migration, and lymphocyte activation pathways, including *ERG, AGAP1, STAP1, ITGA6, PTPRM, C6orf89, TCF12* and *CLEC12A* [5, 66–69]. Genes downregulated in *IGH::DUX4* cells, on the other hand, are implicated in pre-B cell receptor signalling and immune-related pathways.

The transcriptional activity of IGH::DUX4 is highly similar to that of DUX4 protein in which the entire TAD has been knocked out (DUX4-del50) [5]. p300 selectively binds wt DUX4 but not IGH::DUX4, and induction of the rearranged protein does not increase H3K27Ac modifications or chromatin accessibility at its DNA binding sites [5]. These findings suggest that IGH::DUX4 protein is not a pioneer factor, though it is able to initiate transcription and alternative splicing despite harbouring an inactive TAD. Recent studies have highlighted transcriptional cofactors that could account for this neomorphic activity.

Zhang et al. [70] detected recombination signal sequence (RSS) like motifs in IGH::DUX4 targets *ERG*, *CLEC12A*, and *C6orf89*, to which RAG1/2 binding was confirmed by in vitro cleavage assay. Knockdown of RAG1/2 significantly reduced expression levels of alternative transcripts, including *ERG*alt. Li et al. [68] found that the alternative splicing activity of transcription factor *TFC12* is also increased in *DUX4*-r cells, while induction of the fusion protein upregulates *TCF12* expression, suggesting a positive-feedback mechanism. Both RAG1/2 and TCF12 interact with a positively-charged pocket created by the dimerization of two IGH::DUX4 proteins at their homeodomains, disruption of which ceased IGH::DUX4-driven alternative splicing activity. Reduction of IGH::DUX4 activity by TCF12 knockdown suggests that TCF12 is a RAG1/2 cofactor rather than a competitive inhibitor.

IGH::DUX4 does not activate its targets in human embryonic kidney or T-ALL Jurkat cells, leading Campulungo et al. [5] to hypothesize that it interacts with a transcription factor that is preferentially expressed in B-cell precursors. General transcription factor IIi (GTF2I) is the most abundant interactor of IGH::DUX4 in REH cells - an ALL cell line - but not in the other cells types, and CUT&Tag analysis showed selective binding of GTF2I to DUX4-r target genes in REH cells even in the absence of IGH::DUX4. Increased expression of IGH::DUX4 also upregulated GTF2I, indicating positive feedback similar to that of TCF12, while GTF2I knockdown significantly reduced activity of IGH::DUX4 but not wt DUX4.

Further studies are required to ascertain possible interactions of RAG1/2, TCF12 and GTF2I in *DUX4*-r cells, among other potential cofactors, though the presented evidence indicates that all are implicated in IGH::DUX4-induced transcription. Expression of RAG1/2 and TCF12 are positively correlated during T cell receptor (TCR) development [71], suggesting TCF12 cooperates with RAG1/2 during rearrangement of TCR genes, and it is possible that a similar mechanism is aberrantly triggered by *IGH::DUX4* expression in B cell precursors. Differences between wt *DUX4* and *DUX4*-r expression and downstream activity are summarised in Table 1.

**Table 1:** Comparison of features of wild-type DUX4 and DUX4-rearranged proteins.

Feature	Wild-type *DUX4*	*DUX4*-rearrangement
**Genetic Context**	Encoded in the D4Z4 or 10q26 loci as a full-length transcript.	Involves rearrangement with *IGH*, *ERG*, or other gene partners.
**Protein Structure**	Contains a full-length TAD.	Truncated TAD, often replaced by partner gene sequences.
**Transcriptional Activity**	Pioneer factor, recruits p300/CBP for gene activation.	Not a pioneer factor, recruits *RAG1/2, TCF12 and GTF2I* for gene activation and alternative splicing
**Chromatin Impact**	Induces H3K27Ac modifications at its binding sites.	Does not induce H3K27Ac or increase chromatin accessibility.
**Target Genes**	Activates genes essential for early embryogenesis (e.g., *ZSCAN4, DUXA, RFPL4A*).	Activates genes related to cell adhesion, migration, and cancer metastasis (e.g., *ERG, ITGA6, AGAP1*).
**Expression Pattern**	Transiently expressed in cleavage-stage embryos, germline cells and thymus	Aberrantly expressed in B-cell precursors
**Splicing**	Canonical splicing with full-length *DUX4* transcripts.	Initiates alternative splicing in target genes (e.g., *ERG*alt).
**Associated Diseases**	FSHD, advanced-stage solid cancers	B-cell acute lymphoblastic leukaemia
**Immune Evasion**	Downregulates chemokines and MHC class I in advanced-stage solid cancers.	Downregulates immune-related pathways, e.g., cytokine signalling

*TAD* - transactivation domain, *FSHD* - facioscapulohumeral muscular dystrophy.

### Lineage infidelity and immunophenotype

Single-cell analysis has revealed a heterogenous cell maturation profile in *DUX4*-r B-ALL that is not typically observed among other subtypes. While cells with *BCR::ABL1, ETV6::RUNX1* and high hyperdiploid subtypes mainly resemble normal pro-B cells, *DUX4*-r cells appear to evade this ‘maturation block’ and display a broader range of differentiation states, including profiles corresponding to mature B cells [72]. Expression of *NFAT3* is more abundant among early-stage *DUX4*-r cells, while mature cells preferentially express *CEBPA* and *FLT3* [64, 72]. The extent of cell maturation differs among *DUX4*-r cases, leading Brady et al. [64] to delineate *DUX4*-r into *DUX4*-a and *DUX4*-b according to their differential gene expression, with *DUX4*-b corresponding to samples containing mature cell types.

*DUX4*-r leukaemic blasts also demonstrated a high level of lineage infidelity compared to other subtypes, exhibiting characteristics of myeloid (e.g., CD371 expression) and T cells (e.g., CD2 and GATA3 expression), coupled with downregulation of B-cell markers [72, 73]. Expression of CD371 is almost unique to the *DUX4*-r B-ALL [74, 75] and CAR T cell immunotherapy targeting CD371 has been shown to have a potent anti-leukaemic effect on *DUX4*-r cell populations [72].

In a high proportion of *DUX4*-r cases, blasts also undergo monocytic switch during induction therapy, characterised by a decrease in expression of leukaemic markers CD10, CD34, and CD20, and an increase in monocytic markers CD14 and CD33 [73, 75]. Higher expression of CEBPA, FLT3 and TLR10 has also been reported in *DUX4*-r cases with monocytic switching compared to cases without [72, 73], suggesting that it is a feature of mature B cells, while *CEBPA* has been shown to inhibit several B cell regulator genes [76]. No association has been detected between secondary alterations in *DUX4*-r B-ALL and monocytic switching [73].

### Clinical features and outcome of patients with ALL and DUX4-r

*DUX4*-r has an overall frequency of 5–10% of B-cell precursor cases [64]. The frequency of this subtype varies by age with a peak frequency in older children/teenagers [3]. The median age of patients with *DUX4*-r is in the range of 8-13 years, which is significantly higher than other BCP-ALL patients (Table 2), who have a median age of ~5 years old. There is no reported association with white blood cell count at diagnosis or sex. Despite these differences, most paediatric and young adult studies have reported a very good outcome for patients with *DUX4*-r with several of the larger and more recent studies reporting event-free and overall survival rates as high as 95% at 5–10 years (Table 2). Even though the outcome of adults with *DUX4*-r are not quite as high, they are nonetheless very encouraging given the overall poor outcome reported for adults with ALL [77].

**Table 2:** Overview of the demographic features, treatment response and outcome of patients with DUX4-rearranged ALL, and those exhibiting markers associated with DUX4-rearrnangement, from key studies.

Study	Trial	Number of DUX4r cases	Frequency in BCP-ALL^a^	Median age (years)	MRD positive at EOI	Treated as standard risk	Treatment period	Outcome at 5 years
Lilljebjorn et al. [52]	NOPHO-ALL 1992/2000/2008	28	4%	9	NA	NA	1992–2013	4/28 relapsed
Yasuda et al. [3]	ALL202-U	10	14%	19^c^	NA	NA	2002–2009	DFS > 80%
Zhang et al. [2]	St Jude / COG	>50	NA	NA	NA	NA	NA	EFS > 90%
Li et al. [98]	Multiple cohorts	63	5%	NA	NA	NA	NA	Paed OS > 90%; Adult >70%
Schinnerl et al. [74]	AEIOP-BFM 2000/2009/2017	70	6%	10	88%	9%	1999–2023	EFS 89% (10 y)
Ueno et al. [81]	JACLS-ALL02 & TCCSG-L04-16	57	6%	9	NA	NA	2002–2013	EFS 81%
Jeha et al. [99]	St Jude Total Therapy 16	20	4%	8	5%^d^	40%	2007–2017	EFS 95%
Li et al. [61]	MASpore 2003/2010	51	14%	10	78%	NA	2002–2011	OS > 95%
Paietta et al. [7]	ECOG2993	22	8%^b^	26^c^	NA	NA	1993–2005	OS 75%
Brady et al. [64]	Multiple COG & St Jude trials	96	4%	13	NA	NA	NA	OS > 95%
Leongamornlert et al. [57]	UKALL14	8	NA	40^c^	13%	25%	2012–2017	5/8 relapsed
Schwab et al. [6]	UKALL2003	80	5%	9	55%	36%	2003–2011	EFS 95% (10 y)
Yu et al. [8]	TPOG-ALL 2002/2013	26	5%^b^	NA	29%	27%	2002–2020	EFS 83%
**Study**	**Trial**	**Number of cases showing surrogate marker**	**Frequency in BCP-ALL**^**a**^	**Median age (years)**	**MRD positive at EOI**	**Treated as standard risk**	**Treatment period**	**Outcome at 5 years**
Clappier et al. [62]	EORTC-CLG 58951	29 ERGdel	3%	7	100%	0%	1993–2006	EFS > 86% (8 y)
Zaliova et al. [60]	BFM-ALL-2000	60 ERGdel	5%	NA	60%	11%	2000–2006	EFS 90%
Buldini et al. [75]	AIEOP-BFM ALL 2009	160 CD371+	9%	20% ≥10 y	89%	11%	2014–2017	EFS 88%

^a^estimated for many studies form frequency in B-other ALL; ^b^BCR::ABL1 negative cohort; ^c^teenager/young adult or adult cohort; ^d^day 42 MRD. NA, not available.

These excellent survival rates contrast with the relatively poor initial treatment response that has been reported by numerous studies. It is very difficult to directly compare initial treatment response rates across studies due to different induction regimens and variability in the timing and method of assessing measurable residual disease (MRD). Lineage infidelity may also confound immnophenotype-based approaches to detecting MRD that only take into account lymphoid markers, leading to an under-reporting of MRD among *DUX4*-r cases.

Nonetheless, there is a clear trend towards patients with *DUX4*-r responding more slowly to therapy than other patients. For example, in UKALL2003, 55% of patients with *DUX4*-r were MRD positive (≥0.01% at the end of induction, as assessed by PCR) compared to just 21% of patients with *ETV6::RUNX1* [6]. Yoshimura and colleagues recently reported differential drug sensitivity by both age and genetic subtype, which may explain this apparent paradox, finding that older paediatric cohorts and DUX4-r cases may respond better to therapy stages later than induction [78]. However, as *DUX4*-r patients are more likely to be high risk due to their age and have MRD at the end of induction, they are also more likely to have been treated on more intensive treatment arms. Once again, as each trial has different risk stratification criteria, it is difficult to compare studies directly. However, for most studies the proportion of *DUX4*-r treated as standard risk is no more than one-third of cases.

In UKALL2003, only 36% of patients with *DUX4*-r were treated in regimen A (the lowest intensity protocol) compared with 70% of patients with *ETV6::RUNX1* [6]. Therefore, at the moment we do not know if the excellent outcomes reported for patients with *DUX4*-r are because it is an intrinsically chemosensitive subtype, like *ETV6::RUNX1*, or because they were more likely to have received intensive chemotherapy. Few studies have reported outcome by treatment arm, but it is noteworthy that in UKALL2003, only one of the four relapses came from the cohort treated on regimen A [6]. In addition, CD371+ patients treated as standard/medium risk had an EFS of 91% at 5 years compared with 85% for those treated as high risk [75]. It is also currently unknown whether improved outcomes are observed among DUX4-r cases treated with more recently developed therapies, such as the immunotherapeutic Blinatumomab [79].

Even though *IKZF1* deletions have been widely reported as an adverse biomarker [80] several studies have now shown that they are not associated with an inferior outcome among *DUX4*-r patients [60–62, 74]. Although one study has reported an adverse effect of ‘*IKZF1*plus’ [74] – the co-occurrence of *IKZF1* deletions with deletions in *CDKN2A, CDKN2B or PAX5*, or *P2RY8::CRLF2 fusion* [80] – Li et al. [61]. reported an improved MRD response for *DUX4*-r patients with *ERG*del, but this did not translate into a difference in outcome as reported by Schinnerl et al. [59]. Two studies have reported a small number of *DUX4*-r cases with co-occurring *TP53* mutations and relapse rate (two of four cases and four of five cases) [74, 81]. Brady et al. [64] identified two gene expression sub-clusters within *DUX4*-r (*DUX4*-a and -b) with differential outcomes. *DUX4*-a cases had a superior outcome and were enriched for *ERG* and *TBL1XR1* deletions, whereas *DUX4*-b cases were enriched with *NRAS* mutations, *IKZF1* deletions and *KMT2D* mutations [64]. Additional dedicated biomarker studies that integrate treatment information are required to resolve some of these discrepancies and determine which risk factors within *DUX4*-r are clinically relevant.

### Detection

#### Cytogenetics, FISH and SNP array

*DUX4* rearrangement events cannot be detected by conventional cytogenetic approaches. Due to their small size (<10 kb) and typical insertion into the telomeric *IGH* locus, the translocated *DUX4* sequence is not visible by G-banded karyotyping or fluorescence in situ hybridisation (FISH) [52]. Moreover, the repetitive nature of D4Z4/10q26 arrays, in addition to the presence of multiple D4Z4-like sequences across the genome, greatly increases the likelihood of cross-hybridisation by FISH or SNP (single-nucleotide polymorphism) array probes, leading to ambiguous or false-negative findings.

#### RNA-seq

Transcriptomic analysis has proved to be successful for detecting *DUX4*-r cases, and is the approach utilised by 20/26 (77%) studies that have successfully identified them to date. Many have utilized alignment-based tools used to identify fusion breakpoints in RNA-seq data, such as STAR-fusion [82] and FusionCatcher [83]. The unique gene expression profile of *DUX4*-rearranged B-ALL also lends itself to accurate detection using differential gene expression (DEG) analysis. Studies utilising this approach typically employed statistical methods such as DESeq2 [84] to identify significantly upregulated/downregulated genes among cases using RNA-seq data. These are typically complemented by clustering and visualization techniques such as prediction of microarrays (PAM), hierarchical clustering, and t-distributed stochastic neighbour embedding (tSNE) to group samples according to their distinct transcriptional signatures. Machine learning algorithms ALLCatchR [69] and ALLsorts [85] build on these approaches to accurately assign B-ALL subtype, including *DUX4*-r, based on DEG. Both fusion analysis and DEG-based approaches consistently detect *DUX4*-r at a frequency of 5-10% in B-ALL cohorts [1, 2, 7, 52, 57, 59, 61, 63, 64, 74, 86, 87].

#### Short-read whole genome sequencing

Other approaches used to detect *DUX4*-r fusions at the genomic level include short-read whole-genome sequencing (WGS). Leongamornlert et al. [57]. found that the GRIDSS structural variant caller [88] could accurately identify *DUX4*-r fusions, as it employs a contig assembly approach to reconstruct sequences in WGS data around potential breakpoints from reads that show evidence of fusion events (such as splitting or discordant pair mapping), thereby mitigating the impact of off-target read alignments. This approach detected eight *DUX4*-r cases in a cohort of fifty-seven adult B-other ALL (14%).

The recently developed Pelops [89] tool is specifically designed to detect *DUX4*-r fusions. Pelops identifies read pairs that span the fusion junction between *DUX4* and other genomic loci, such as *IGH*, in WGS data. These spanning read pairs are normalized using a spanning read pairs per billion total reads (SRPB) metric, which adjusts for differences in sequencing depth across samples to ensure accurate comparison. Pelops successfully identified all *DUX4*-r cases for which there was orthogonal evidence in a validation cohort [1], totalling 60/210 (28.6%) paediatric B-other cases.

#### Long-read sequencing

Long-read approaches have been used to successfully characterise the endogenous *IGH::DUX4* fusion event in the NALM6 cell line [53]. By barcoding short reads that belong to the same high molecular weight DNA fragments using the 10x Genomics Chromium platform, 8 kb and 4.5 kb contigs could be assembled, which included the *IGHM* region, Eμ enhancer, translocated 10q26 *DUX4* sequence, and other *IGH* sequences near the breakpoints.

PacBio and Oxford Nanopore Technology (ONT) long-read platforms can directly produce reads >10 kb in length, mitigating the potential for poor mapping at the *IGH* locus. Yasuda et al. [3] performed whole-genome sequencing on the NALM6 cell line using a PacBio single-molecule real-time (SMRT) platform, producing reads with a mean length of 13.3 kb [3]. Three reads successfully mapped the full and partial copy of chr10 *DUX4* present at the *IGH* locus, confirming the *IGH::DUX4* rearrangement reported in this cell line by previous studies. In the first published study that used ONT sequencing to subtype B-ALL cases, Kato et al. [90] used a targeted approach known as adaptive sampling to identify fusion breakpoints in tumour samples whose mean N50 was 11 kb. For detection of *DUX4*-r B-ALL, the *DUX4, IGH* and *ERG* loci were sequenced and subjected to structural variant (SV) detection using Nanomonsv [91], while de novo sequencing assembly was used for comprehensive breakpoint ascertainment. Three *IGH::DUX4* cases were identified among thirteen B-ALL cases, all of which were found to carry *ERG*del.

#### Indirect detection methods

As *ERG*del is detectable by multiplex ligation-dependent probe amplification (MLPA), SNP array, and breakpoint-specific polymerase chain reaction (PCR) [92], it is often relied on as a surrogate marker for *DUX4*-r in B-ALL cases. Probe signal may be too weak to detect this alteration when it occurs subclonally, though qPCR and amplicon-sequencing have been shown to somewhat overcome this limitation [63]. However, *ERG*del has been observed in a small number of cases that do not harbour a *DUX4* rearrangement and is absent in around 40–60% of *DUX4*-r cases, limiting its reliability for diagnostic purposes [63]. *ERG*alt expression has also been suggested as a surrogate marker, but levels vary widely between cases and it has also been observed in low amounts among other B-ALL subtypes [2, 63].

Immunophenotyping has demonstrated much greater reliability in detecting *DUX4*-r cases, due to the non-lymphoid markers that are almost uniquely expressed in this subtype. In particular, flow cytometry of CD371 – an approach typically employed for the detection of myeloid malignancies – identified all but one *DUX4*-r in a cohort of 46 cases, 91.3% of which showed strong expression of this marker at diagnosis [74]. A subfraction of blasts showed weak *CD371* expression in six instances of other subtypes, namely two high hyperdiploid, one near haploid, one *BCR::ABL1* and two undefined B-other cases. Similarly, Buldini et al. [75]. found that 27/28 (96.4%) of B-ALL cases carrying *DUX4*-r fusions were CD371-positive at diagnosis, compared with 7/387 (1.8%) of cases of other subtypes, indicating very high sensitivity and specificity for CD371 detection as a marker for *DUX4*-r B-ALL.

Moreover, combined expression of CD371 and CD2 is thought to exclusively occur in *DUX4*-r cases [59, 74, 75], though, as CD2 is only expressed in 50-75% of samples, detection of both markers is not as sensitive as the detection of CD371 alone. In addition, Buldini et al. [75] found that CD371 had a sensitivity of 93% for predicting the myelomonocytic lineage infidelity among B-ALL cases at diagnosis, compared to 56% for CD2.

Immunohistochemistry targeting the N-terminus of the *DUX4*-r fusion protein has recently been suggested as a reliable approach for diagnosis. Among six B-ALL cases for which there was RNA-seq or immunohistological evidence of *DUX4*-r, Siegele et al. [93] reported strong nuclear staining with an N-terminus DUX4 antibody, while three additional cases that were known to be negative for *IGH::DUX4* showed no evidence of staining. Further studies may be required to ascertain the specificity of this approach, given that low levels of *DUX4* expression have been observed in *DUX4*-r negative B-ALL cases [63]. However, it is unclear whether this indicates the presence of translatable DUX4 mRNA or is due to technical artifacts such as cross-sample contamination.

#### Comparing *DUX4*-r detection methods

Despite technical differences between direct detection approaches, the reported frequency of *DUX4*-r cases remains consistent across studies (5-10%), with exceptions observed only among smaller studies. Given the relative lack of studies that have utilised DNA-seq data compared to RNA-seq, a robust comparison of the sensitivities of the corresponding detection methods is not yet feasible. However, on the basis of current literature, there is no evidence to suggest that optimised short-read WGS approaches (e.g., GRIDSS, Pelops) have greater sensitivity than RNA-seq and vice versa, while the additional utility of long-read technologies currently seems to be limited to more accurate resolution of breakpoints, particularly at partner gene loci.

A disadvantage of DEG analysis is that is it only applicable in the context of B-ALL, as it depends on comparison of the *DUX4*-r gene expression profile (GEP) with that of other B-ALL cases. Similarly, CD371 can only be used as a surrogate marker for *DUX4*-r relative to its expression among cases of other B-ALL subtypes. By contrast, gene fusion analysis (using both short-read/long-read WGS and RNA-seq) can be used to detect *DUX4*-r B-ALL even where diagnostic information is unavailable, and can also detect *DUX4* rearrangements occurring in non-leukaemic cancers. For example, the Pelops tool has successfully identified *DUX4* fusion events in other cancer types across Genomics England cohorts, many of which were not previously known to be associated with *DUX4* rearrangements (Alona Solinsky, personal communication, January 2025).

On the other hand, clinically relevant differences in GEP associated with cell maturation profiles are not detectable by gene fusion analysis and there is no evidence that different *DUX4* partner genes in B-ALL contribute to differential gene expression among *DUX4*-r cases. Moreover, while CD371+ is associated with good long-term outcomes in B-ALL, almost all *DUX4*-r cases show strong expression of this antigen, so it cannot be used to assess outcomes between *DUX4*-r cases. DEG analysis may therefore be more informative for the evaluation of outcomes within this subtype than gene fusion analysis and CD371 flow cytometry.

The costs associated with each detection method are an important consideration in both clinical and research settings. High-throughput RNA-seq is more affordable than both short-read and long-read WGS. Given the potential to differentiate between the two *DUX4*-r groups (*DUX4*-a and *DUX*4-b) from RNA data but not WGS, it is the most cost-effective option for both *DUX4*-r detection and downstream clinical analyses. CD371 flow cytometry is relatively cheap, highly accessible and faster than sequencing [94], which, combined with its high sensitivity and specificity for *DUX4*-r detection, makes it the best option in clinical settings where access to more expensive sequencing equipment is limited.

Long-read WGS is currently one of the most costly options, with PacBio platforms being more expensive than ONT, which are capable of producing longer read lengths [95]. ONT platforms have a higher sequencing error rate, which may impact variant calling; however, these have largely been mitigated by improved basecalling algorithms [96]. In addition, ONT platforms generate data in real-time during runs, leading to improved efficiency over PacBio and short-read platforms. Further optimisations of ONT platforms are likely to reduce the per-sample cost of long-read WGS below that of short-read alternatives [97]. Long-read platforms can also generate RNA-seq data, though the utility of this in the context of B-ALL is yet to be investigated.

## Conclusions

The unique biology of the *DUX4* transcription factor and its rearrangements underpins its roles in both normal developmental processes and disease. In B-ALL, *DUX4*-rearranged cases occur at a frequency of 5-10%, and exhibit a distinct transcriptional profile driven by a truncated DUX4 protein, which recruits alternate cofactors for gene activation and splicing. The downstream consequences of its unusual activity are demonstrated by aberrant expression of non-lymphoid markers (e.g., CD371), unique structural variation (*ERG*del), a broad cell maturation profile, and a higher incidence of MRD at the end of induction therapy. Despite these features, the overall prognosis for *DUX4*-rearranged B-ALL patients remains favourable, with event-free and overall survival exceeding 80% in most paediatric cases. Advancements in sequencing technologies have the potential to both markedly improve and simplify detection of this notoriously cryptic subtype, leading a more comprehensive genomic profiling and a robust understanding of the mechanisms behind *DUX4* rearrangement.

### Future work

- Understand the mechanisms by which *DUX4* rearrangements occur, and the genomic, transcriptional and biological features shared among its many identified gene partners.
- Understand how *DUX4*-r activity - and in particular its capacity for alternative splicing - leads to B-cell lineage development, infidelity and the evasion of maturation blocks typically not observed in B-ALL.
- Comparative studies of *DUX4* rearrangements among other cancer types, given preliminary findings that *DUX4* rearrangements can occur in several other malignancies beyond B-ALL and *CIC*-r sarcoma, which may reveal shared oncogenic pathways.
- Reach an international consensus on the best approaches to *DUX4*-r detection at diagnosis, with a focus on those that can be adopted in clinical settings without access to cutting-edge sequencing technology.
- Improved risk stratification and prognosis using chemotherapy and, more recently, developed therapies such as Blinatumomab.

## Data Availability

Data sharing not applicable to this article as no datasets were generated or analysed during the current study.
